# Natural and Synthetic Polymer Scaffolds Comprising Upconversion Nanoparticles as a Bioimaging Platform for Tissue Engineering

**DOI:** 10.3390/molecules27196547

**Published:** 2022-10-03

**Authors:** Ekaterina M. Trifanova, Maria A. Khvorostina, Aleksandra O. Mariyanats, Anastasia V. Sochilina, Maria E. Nikolaeva, Evgeny V. Khaydukov, Roman A. Akasov, Vladimir K. Popov

**Affiliations:** 1 Institute of Photon Technologies of Federal Scientific Research Centre “Crystallography and Photonics” of Russian Academy of Sciences, 108840 Moscow, Russia; 2 LLC “Syntol”, 127434 Moscow, Russia

**Keywords:** tissue engineering, collagen, PLGA, hyaluronic acid, 3D printing, electrospinning, upconversion nanoparticles, bioimaging

## Abstract

Modern biocompatible materials of both natural and synthetic origin, in combination with advanced techniques for their processing and functionalization, provide the basis for tissue engineering constructs (TECs) for the effective replacement of specific body defects and guided tissue regeneration. Here we describe TECs fabricated using electrospinning and 3D printing techniques on a base of synthetic (polylactic-co-glycolic acids, PLGA) and natural (collagen, COL, and hyaluronic acid, HA) polymers impregnated with core/shell β-NaYF_4_:Yb^3+^,Er^3+^/NaYF_4_ upconversion nanoparticles (UCNPs) for in vitro control of the tissue/scaffold interaction. Polymeric structures impregnated with core/shell β-NaYF_4_:Yb^3+^,Er^3+^/NaYF_4_ nanoparticles were visualized with high optical contrast using laser irradiation at 976 nm. We found that the photoluminescence spectra of impregnated scaffolds differ from the spectrum of free UCNPs that could be used to control the scaffold microenvironment, polymer biodegradation, and cargo release. We proved the absence of UCNP-impregnated scaffold cytotoxicity and demonstrated their high efficiency for cell attachment, proliferation, and colonization. We also modified the COL-based scaffold fabrication technology to increase their tensile strength and structural stability within the living body. The proposed approach is a technological platform for “smart scaffold” development and fabrication based on bioresorbable polymer structures impregnated with UCNPs, providing the desired photoluminescent, biochemical, and mechanical properties for intravital visualization and monitoring of their behavior and tissue/scaffold interaction in real time.

## 1. Introduction

Modern biocompatible materials of both natural and synthetic origin, in combination with advanced methods for their processing and functionalization, make it possible to form tissue engineering constructs (TECs), providing hybrid tissue equivalent fabrication for the effective replacement of specific defects in human and animal organs [1,2,3,4] as well as guided regeneration of their damaged or missing fragments [5]. One of the key elements of such biomedical products is three-dimensional (3D) bioresorbable scaffolds, which enable efficient attachment, differentiation, and proliferation of the required cell type cultures [6].

The most important criteria for choosing both starting materials and the architectonics of the scaffolds are the maximum matching of their physicochemical, biochemical, and mechanical characteristics with the analogous parameters of the native tissue to be replaced or regenerated as well as the negligible (in principle, zero) severity of the body’s immune response to their implantation [7]. Tissue equivalents based on implantable polymeric scaffolds should have physical–biochemical characteristics that are as close as possible to their analogues of the recipient’s native tissues. Ideally, the scaffold should not cause pronounced acute inflammation of the surrounding tissues at the site of its implantation and should not initiate a fibrous capsule formation at the scaffold/living tissue interface, thereby ensuring the absence of chronic inflammation [8].

The most common materials for scaffold fabrication are biocompatible synthetic polymers (aliphatic polyesters, polyanhydrides, polyurethanes, etc. [9]) as well as polymers of natural origin (biopolymers), such as collagen (COL) and hyaluronic acid (HA), which are the key components of the extracellular matrix [10]. Collagen has excellent biocompatibility, controllable bioresorption, and low immunogenicity. It is actively used today in various TECs both in pure form [11] and in mixtures with various synthetic polymers, such as polycaprolactone [12], polylactide [13], polylactoglycolide [14], and polyethylene glycol [15]. Collagen scaffolds are used in the tissue engineering of nervous, bone, and cartilage tissues as well as tissues of tendons, ligaments, blood vessels, and skin [16]. Other natural biopolymers, such as hyaluronic acid and its different modifications (including HA-based acrylated compounds [17]), are intensively used in tissue engineering of cartilage structures, in particular, promoting active proliferation and migration of chondrocytes [6].

Currently, there is a huge variety of different physicochemical methods and technologies for the manufacture of bioresorbable polymer scaffolds that mimic the extracellular matrix [18]. In our present study, we have used three of them: electrospinning and two types of three-dimensional (3D) printing, namely anti-solvent extrusion 3D printing and extrusion 3D printing with simultaneous photocuring.

Electrospinning is a common method for manufacturing non-woven fibrous structures from various materials [19]. During this process, thin (from 0.1 to 10 µm in diameter) filaments are formed from a polymer solution or melt-injected through the nozzle under the action of high voltage (ca. 10–20 kV) electrostatic field, which are randomly collected on the “ground” electrode substrate (usually stainless steel or aluminum plate) [20]. The resulting non-woven fibrous and biocompatible structure has a high surface to volume ratio with porosity up to 97% [21], promoting effective cell adhesion and proliferation [22]. During electrospinning, intra- and intermolecular bonds between fibers are practically not formed, which leads to a loss of structural stability of the scaffold in an aqueous solution. To solve this problem, various chemical, photochemical, and physical methods are used today [23,24]. Chemical cross-linking of collagen scaffolds is carried out by adding various cross-linking agents [25]. The choice of cross-linking agent is made taking into account the reactive group present, usually an amino acid chain side, and the appropriate environmental conditions (pH, temperature, and solvents) to preserve native collagen structure. The mechanism of collagen cross-linking in an acidic environment includes the reaction of carboxylic acid groups with glutamic and aspartic acids and epoxy groups of 1,4-butanediol diglycidyl ether. Crosslinking at acidic pH values from 4.5 to 6.0 occurs via a reaction mechanism in which the epoxy groups become protonated, followed by a nucleophilic attack on the carboxylate anion to form an ester bond [26].

3D printing is one of the most promising methodologies for the precise and reproducible fabrication of TEC scaffolds [27]. Among the main 3D printing methods that exist today, extrusion 3D printing should be highlighted [28]. During extrusion printing, viscous polymer solutions are forced out of a nozzle and solidified by different means on a support platform [29]. The required structures are formed by sequential layer-by-layer extrusion of the material, following a predetermined trajectory built using three-dimensional computer modeling. All this allows to create complex structures of a predetermined architectonics [30]. Today, a large number of studies are devoted to the search and development of low-temperature methods for the TEC scaffold fabrication [31]. The main challenge of 3D printing methods is in providing the necessary conditions for the curing process of viscous liquids on a substrate. This may involve the initiation of photocurable composition (e.g., methacrylated HA), polymerization by the laser radiation, or phase separation in the polymer–solvent–antisolvent systems [32,33]. These processes can occur at near-room temperatures that do not reach the temperature of material destruction, which guarantees the preservation of their physical–chemical and biochemical properties.

Conventional methods for in vivo analyzing the results of TECs implantation, their behavior in surrounding tissues, and the immune response require, as a rule, the euthanasia of laboratory animals to extract the implanted scaffolds with adjacent tissues for their detailed morphological and histochemical studies [34]. At the same time, there are many non-invasive approaches to intravital visualization of foreign materials and structures inside the body that have been successfully developed and effectively used over the past decades [35]. These include X-ray and computed tomography [36], magnetic resonance imaging [37], positron emission [38], and ultrasound [39] techniques as well as various options for multimodal imaging [40]. However, some challenges can appear. As noted above, close-to-ideal hybrid tissue equivalents based on implantable polymeric scaffolds should have physical–biochemical characteristics that are as close as possible to their analogues of the recipient’s native tissues so that it is almost impossible to distinguish them from the surrounding living tissues via usual methods [41].

One of the promising approaches to solving this problem is the use of upconversion nanoparticles (UCNPs), which have unique optical properties, including chemical and photostability, narrow band and large luminescence shifts relative to excitation light [42,43]. Due to their inorganic crystalline matrix doped with lanthanide ions, UCNPs can transform NIR excitation into radiation of UV, visible, and NIR spectrum range with a high (up to 10%) integral conversion efficiency [44]. This could successfully adapted for various biophotonics applications, including bioprinting [45] and photodynamic therapy [46]. Conversion efficiency of the upconversion nanoparticles can be significantly increased by coating with an inert shell [47,48]. Today UCNPs are used in various diagnostic systems, including visualization of tumors, the lymphatic system, and blood vessels [42] as well as for optogenetic control of neuronal activity [49]. Recently, 3D silk fibroin scaffolds implemented with Yb^3+^/Er^3+^ UCNPs were successfully utilized for subcutaneous near-infrared optical imaging into mice [50]. Another type of core/shell UCNPs co-doped with Yb^3+^/Tm^3+^ were used to induce biomacromolecules release from hydrogels due to conversion of NIR photons into UV and triggering gel–sol transition [51]. Various UCNPs can also be incorporated into electrospun fibrous polymer matrices for broad photonic applications [52,53].

Within this context, our research focuses on development of the technological platform enabling so-called “smart scaffold” fabrication based on bioresorbable polymer structures impregnated with upconversion nanoparticles as well as on the study of their photoluminescent, biochemical, and mechanical properties for intravital photoluminescent visualization and monitoring of exogenous body materials in a real-time manner.

## 2. Results

### 2.1. UCPNs core/shell β-NaYF_4_:Yb^3+^:Er^3+^/NaYF_4_

**Figure 1:**
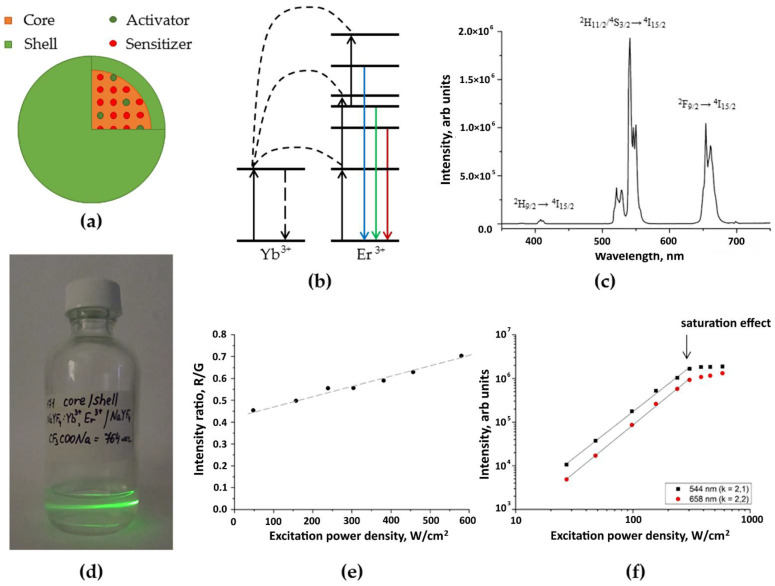
Characterization of upconversion core/shell β-NaYF_4_:Yb^3+^:Er^3+^/NaYF_4_ nanoparticles: (**a**) schematic design of core/shell β-NaYF_4_:Yb^3+^:Er^3+^/NaYF_4_ UCNPs; (**b**) energy level diagram of the UCNPs; (**c**) photoluminescence spectrum of the UCNPs; (**d**) photograph of the UCNPs under excitation at 976 nm; (**e**) dependence of the ratio of the intensity of the red luminescence band at a wavelength of 658 nm to that of the green one at a wavelength of 544 nm on the power density of exciting laser radiation for UCNPs; (**f**) dependences of the photoluminescence intensity on the power density of exciting radiation on a double logarithmic scale at wavelengths of 544 and 658 nm for the UCNPs.

Upconversion core/shell β-NaYF_4_:Yb^3+^:Er^3+^/NaYF_4_ nanoparticles (Figure 1a) were synthesized and carefully evaluated before entrapment into the scaffolds. The diagram of the energy levels of these UCNPs and the photoluminescence spectra corresponding to them are shown in Figure 1b,c. Figure 1f shows the dependences of the photoluminescence intensity on the power density of exciting radiation on a double logarithmic scale at wavelengths of 544 and 658 nm in detail. Figure 1e shows that the ratio of the intensities of the red peak at a wavelength of 658 nm to the intensity of the green peak at a wavelength of 544 nm (R/G ratio) grows with increasing power density of the exciting radiation. An increase in radiation power density leads to the activation of higher Er^3+^ levels (^4^G_11/2_ и ^4^G_7/2_), from which a nonradiative transition to the ^4^F_9/2_ level is more probable than a transition to the ^2^H_11/2_/^4^S_3/2_ levels [54]. These typical photoluminescence properties could be used as fingerprints to indicate the UCNPs and control their state in the current microenvironment.

### 2.2. Optimization of The Scaffolds’ Mechanical Properties

**Figure 2:**
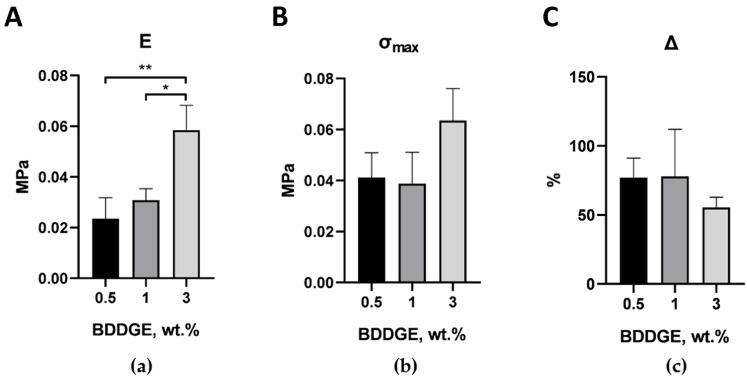
Tensile test results for ELS COL samples immersed in isopropanol with 15 wt.% BDDGE for 6 days and in PBS for 1 day: (**a**) Young’s modulus, (**b**) tensile strength, (**c**) maximum elongation. * *p* < 0.05, **p < 0.01 in Student’s *t*-test; data are the mean ± SD of at least 3 replicates.

Mechanical tests of scaffolds without the UCNPs were carried out to determine the mechanical properties of the initial structures. The mechanical properties of 3D PLGA and 3D HAGM structures had been studied previously for distilled water and PBS at 37 °C [55]. Briefly, 3D PLGA scaffolds demonstrated Young’s moduli values of 2.2 MPa and 6.3 MPa, respectively, while those of the 3D HAGM scaffolds were 0.2 MPa and 0.6 MPa, respectively. In the current research, we evaluated the Young’s modulus, tensile strength, and maximum elongation for ELS COL and ELS PLGA samples. Additionally, to increase the efficiency of the process of chemical crosslinking of the structure of collagen scaffolds following electrospinning, different amounts (0.5, 1, and 3 wt.%) of BDDGE were added to the initial composition with 4 wt.% collagen in HFIP. After electrospinning, the formed scaffolds were carefully removed from the collecting electrode foil and placed in Petri dishes, where they were kept in isopropanol with 15 wt.% BDDGE for 6 days. Then, the first half of the samples was placed directly into PBS for 1 day. The second half of the samples was pre-dried before being placed into PBS for 1 day to remove all the remaining isopropanol in order to understand how the properties of the scaffold change after a long stay in the wet state. Figure 2 shows the results of mechanical tests of collagen scaffolds with different content of BDDGE in the original composition.

**Figure 3:**
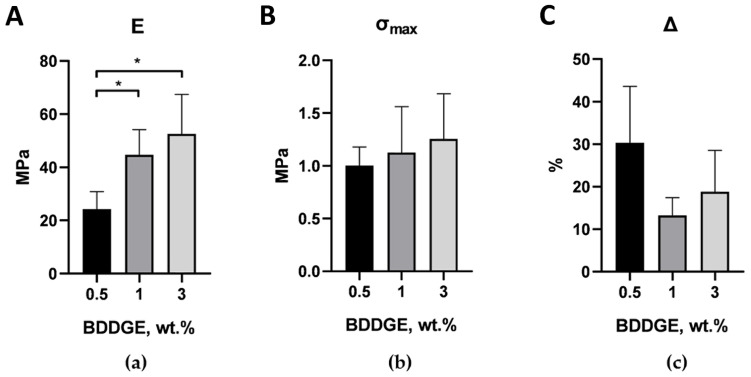
Tensile test results for dry ELS COL samples immersed in isopropanol with 15 wt.% BDDGE for 6 days, dried and immersed in PBS for 1 day: (**a**) Young’s modulus, (**b**) tensile strength, (**c**) maximum elongation. * *p* < 0.05 in Student’s *t*-test; data are the mean ± SD of at least 3 replicates.

The samples with BDDGE contents of 0.5 and 1 wt.% had similar (within the standard error) mechanical characteristics. Samples with 3 wt.% BDDGE had the highest tensile strength and the smallest value of Δ (maximum elongation); however, only differences in tensile strength were statistically significant. For pre-dried ELS COL samples (Figure 3), a similar pattern was observed: with an increase in the concentration of BDDGE in the initial solution, the elastic modulus gradually increased and the value of Δ gradually decreased. It is important that the tensile strength of pre-dried samples was an order of magnitude greater in comparison to the non-dried samples, and the maximum elongation was several times less. This simple step can greatly change the mechanical properties of the scaffolds and could be a tool for creation scaffolds with desirable biomechanics. The pre-dried ELS COL samples with 1 wt.% BDDGE were chosen for further experiments.

**Figure 4:**
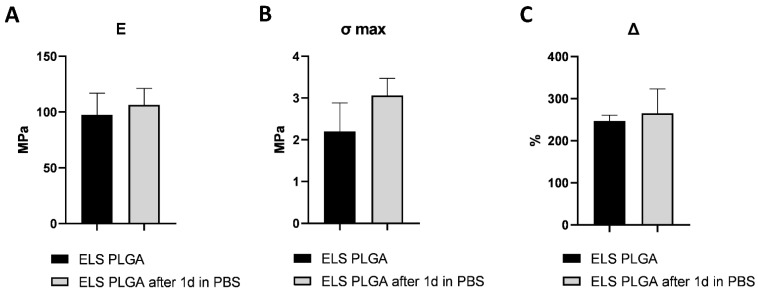
Tensile test results for ELS PLGA samples dried and immersed in PBS for 1 day: (**a**) Young’s modulus, (**b**) tensile strength, (**c**) maximum elongation; data are the mean ± SD of at least 3 replicates.

The results of tensile tests of ELS PLGA samples are presented in Figure 4. ELS PLGA samples immersed in PBS for one day shrank by about 20% and became denser compared to the original samples. In addition, samples immersed in PBS had fewer defects (they did not delaminate during a tensile test) and were more durable. The elongation at break for both types of samples was similar; however, during testing, it was seen that the elongation at tensile strength maximum (without partial breaks due to delamination) in the original samples was much lower, at –5% versus 128% for the immersed ones.

### 2.3. UCNP-Loaded Polymer Scaffolds Formation

**Figure 5:**
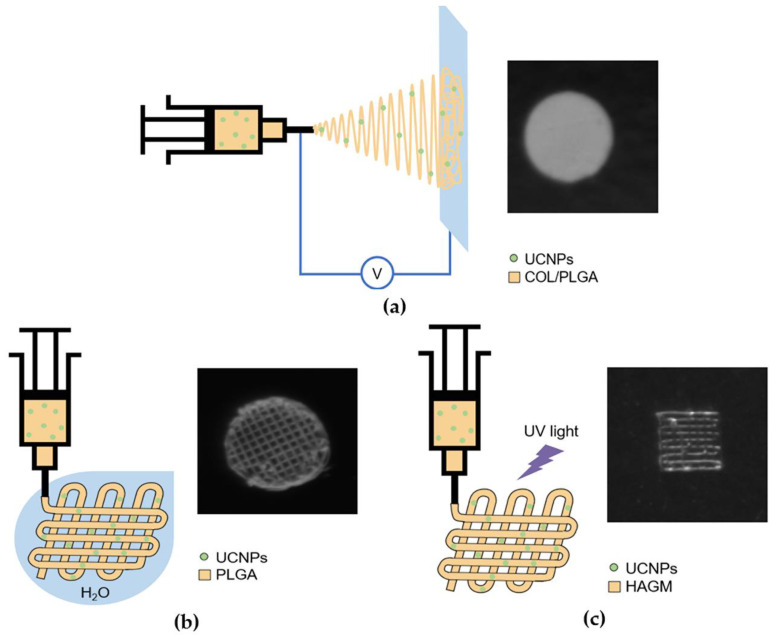
Schematic illustration of custom-build experimental setup for (**a**) electrospinning of polymer solutions; (**b**) anti-solvent extrusion 3D printing; (**c**) extrusion 3D printing with simultaneous photocuring and corresponding photoluminescent imaging of scaffolds.

We produced eight types of UCNP-loaded scaffolds, including ELS COL, ELS PLGA, 3D PLGA, and 3D HAGM scaffolds with concentrations of 0.1 mg and 1 mg of UCNPs per 100 mg of polymer (0.1% and 1%, respectively) (Figure 5). At the stage of preparation of the initial composition, UCNPs were added to the polymer solution (1 mL) in appropriate concentrations. To increase the efficiency of the process of chemical cross-linking of the structure of ELS COL scaffolds, 1 wt.% BDDGE was added to the initial composition with 4 wt.% collagen in HFIP. After electrospinning, the formed UCNP-loaded collagen scaffolds were also immersed in isopropanol with 15 wt.% BDDGE for 6 days. Additionally, 3D HAGM scaffolds were irradiated at a wavelength of 450 nm for 30 min. ELS PLGA and 3D PLGA scaffolds were used without additional processing. The study of the microstructure and morphology of the surfaces of the scaffolds did not reveal significant differences between 0.1% and 1% of UCNPs; however, the luminescent signal was significantly higher for 1% UCNP loading (Figures S1–S4), so we focused on 1% UCNP samples for further research.

**Figure 6:**
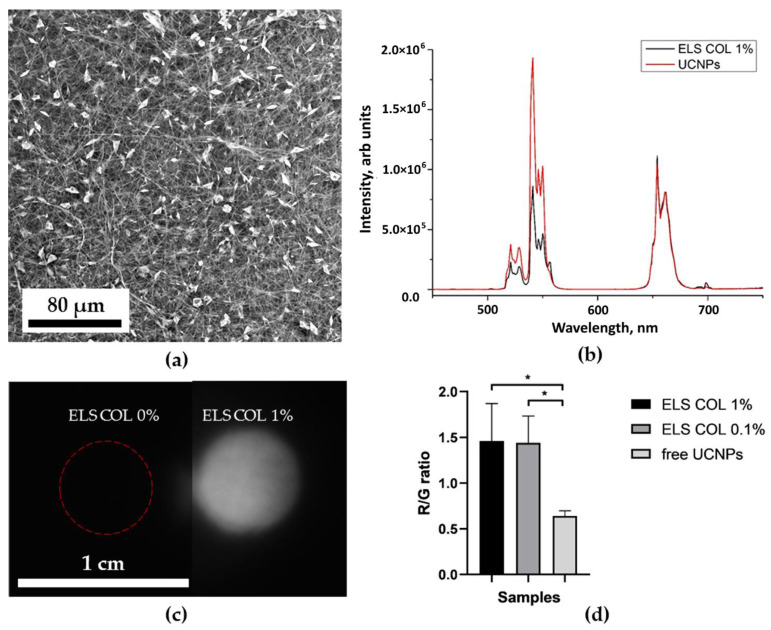
Electrospun collagen scaffold characterization: SEM image (**a**); normalized photoluminescence spectra of the UCNPs and the UCNPs included in ELS COL scaffolds (**b**); photographs of ELS COL scaffolds with 0 (control) and 1 mg of UCNPs per 100 mg of polymer at 976 nm (**c**). Ratio of the intensity of the red peak at 658 nm to the intensity of the green one at a wavelength of 544 nm of UCNPs and ELS COL scaffolds (0.1 and 1% UCNPs) * *p* < 0.05 in Student’s *t*-test (**d**).

**Figure 7:**
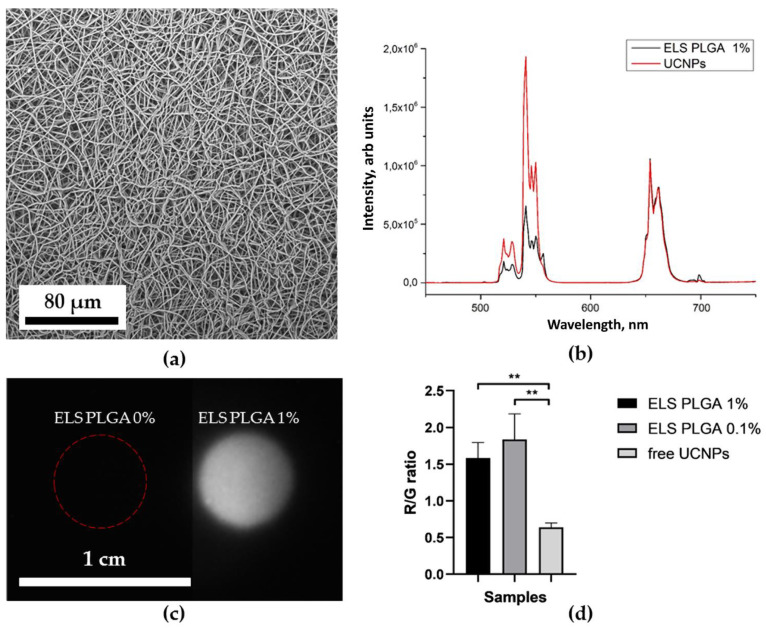
Electrospun polylactic-co-glycolic acid scaffolds: SEM image (**a**); normalized photoluminescence spectra of the UCNPs and the UCNPs included in ELS PLGA scaffolds (**b**); photographs of ELS PLGA scaffolds with 0 (control) and 1 mg of UCNPs per 100 mg of polymer at 976 nm (**c**). Ratio of the intensity of the red peak at 658 nm to the intensity of the green at a wavelength of 544 nm of UCNPs and ELS PLGA scaffolds (0.1 and 1% UCNPs) ** *p* < 0.01 in Student’s *t*-test (**d**).

**Figure 8:**
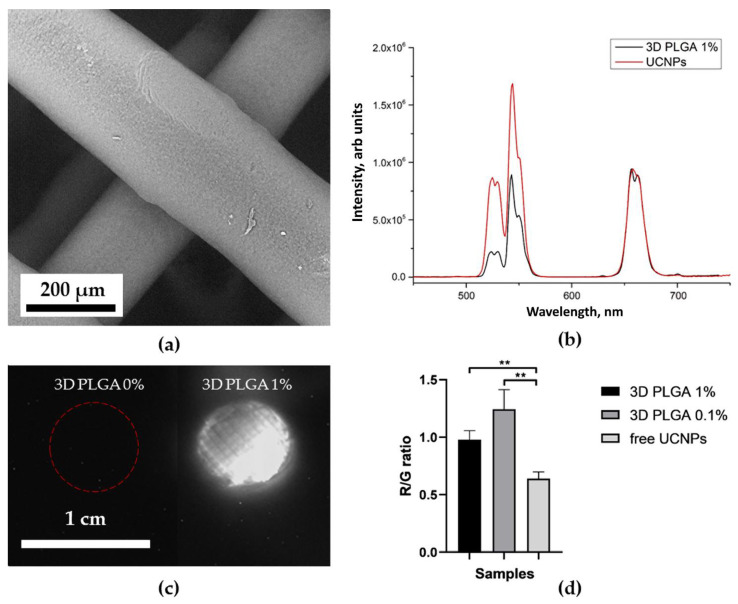
SEM image of 3D-printed polylactic-co-glycolic acids scaffolds (**a**); normalized photoluminescence spectra of the UCNPs and the UCNPs included in 3D PLGA scaffolds (**b**); photograph of 3D PLGA scaffolds with 0 (control) and 1 mg of UCNPs per 100 mg of polymer at 976 nm (**c**). Ratio of the intensity of the red peak at 658 nm to the intensity of the green at a wavelength of 544 nm of UCNPs and 3D PLGA (0.1 and 1% UCNPs) ** *p* < 0.01 in Student’s *t*-test (**d**).

**Figure 9:**
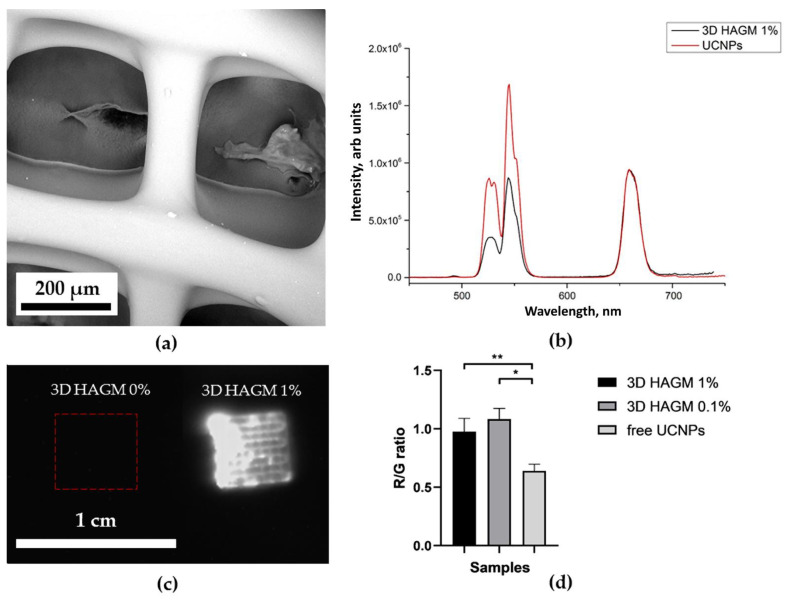
SEM image of 3D-printed HAGM scaffolds (**a**); normalized photoluminescence spectra of the UCNPs and the UCNPs included in 3D HAGM scaffolds (**b**); photograph of 3D HAGM scaffolds with 0 and 1 mg of UCNPs per 100 mg of polymer at 976 nm (**c**). Ratio of the intensity of the red peak at 658 nm to the intensity of the green at a wavelength of 544 nm of UCNPs and 3D HAGM scaffolds (0.1 and 1% UCNPs) * *p* < 0.05, ** *p* < 0.01 in Student’s *t*-test (**d**).

Figure 6b, Figure 7b, Figure 8b and Figure 9b show the characteristic photoluminescence spectra of UCNP-loaded scaffolds compared to free UCNP spectrum. The quenching of the photoluminescence of impregnated UCNPs could be explained by their specific interaction with the polymer macromolecules and by the light absorption and scattering within this scaffold. The intensity of the photoluminescence of impregnated UCNPs also decreased when passing through the polymer surrounding the nanoparticles (Figure S5). Therefore, the amplitude of the detecting signal was lower than that of free UCNPs. The ratio of the intensities of the red peak at a wavelength of 658 nm to the green peak at a wavelength of 544 nm was higher for 3D HAGM scaffolds than the others, indicating more intense nonradiative relaxation of the ^2^H_11/2_/^4^S_3/2_ excited states into the ^4^F_9/2_ state. The differences between the spectra can be explained by interaction of scaffold polymer molecules with UCNPs and optical properties of the scaffold since the polymers scatter and transmit light differently. The HAGM film was less translucent in the green range than in the red and IR ranges (Figure S5); therefore, the peaks are shifted to a longer wavelength region (409 nm to 414 nm, 544 to 549 nm). The received transmittance dependency also explains the low radiation intensity of nanoparticles inside the HAGM scaffolds.

For all types of scaffolds, a statistically significant difference was found in the R/G ratio between free UCNPs and UCNPs impregnated into scaffolds. In addition, a decrease in the concentration of UCNPs (from 1 to 0.1%) did not lead to statistically significant changes in the R/G ratio, which indicated the sensitivity of the method.

### 2.4. Optical Properties of UCNP-Loaded Polymer Scaffolds

Figure 6c, Figure 7c, Figure 8c and Figure 9c show photographs of UCNP-loaded samples obtained with the imaging system that uses a scanning laser beam at a wavelength of 976 nm to pump samples. All studied samples are clearly visible and can be well defined. The photographs show that the UCNPs are evenly distributed over the samples. Table S1 presents the lifetimes of photoluminescence at different wavelengths of nanoparticles recorded for free UCNPs (dry placed onto a glass slide), UCNPs resuspended in water and polymer-impregnated UCNPs. The photoluminescence lifetime of nanoparticles in collagen scaffolds was shorter than that of free nanoparticles. UCNPs in collagen and polylactic-co-glycolic acids scaffolds had similar photoluminescence lifetimes. However, nanoparticles in the HAGM samples had the shortest lifetime in comparison to the photoluminescence lifetimes of UCNPs in water. Changes in lifetime could be explained by non-radiative interactions (quenching) of polymer molecules with rare-earth ions in UCNPs. The polymeric molecules within the scaffold have different effects on the luminescent transitions of trivalent erbium ions in UCNPs involving in non-radiative deactivation pathways. There are clear differences between dry UCNPs and UCNPs resuspended in water [56].

### 2.5. Release of UCNPs

We carried out the release of UCNPs from the scaffold into the aqueous medium to mimic the biodegradation of UCNPs-loaded TECs in vivo. We demonstrated that the spectrum of released UCNPs (extract of ELS COL 1%) has a higher R/G ratio compared to the spectrum of UCNPs inside the ELS COL 1% scaffold (Figure S6b) which is similar to the ratio for UCNPs in PBS (Figure S6a). The increase in the ratio can be explained by the interaction of UCNPs with water molecules [56]. The interaction of Er^3+^ ions with H_2_O molecules leads not only to luminescence quenching, but also stimulates a nonradiative transition, ^4^I_13/2_ → ^4^F_9/2_, which causes the increase in the red peak at 658 nm. Released UCNPs had a similar R/G ratio to free UCNPs (Figure S6c), allowing in vitro release tracking. Ratio change within the time can be used to determine the degree of degradation of the scaffold inside the body to indicate the release of UCNPs from the scaffold. This could be performed in combination with analyzing the area around the scaffolds to track the released UCNPs. Moreover, the power density of the excitation light within irradiated area could be calculated using the Mie scattering data and the specific optical parameters of the biotissue [57].

### 2.6. Cytotoxicity

**Figure 10:**
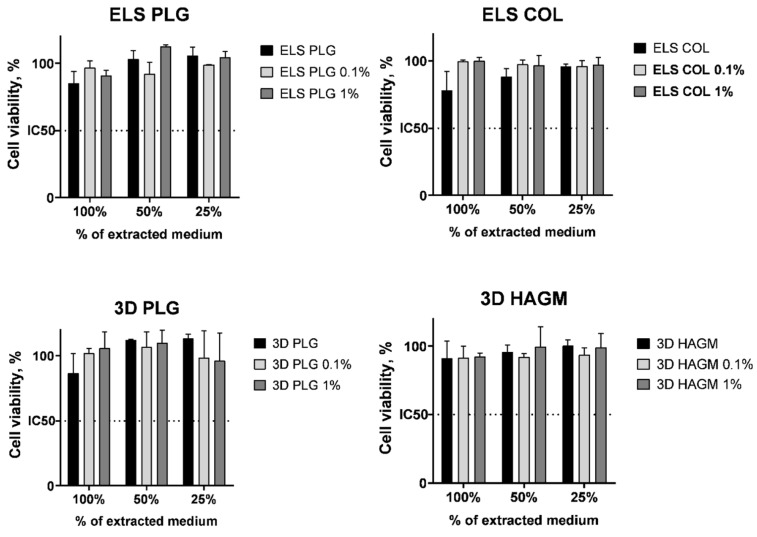
The viability of Bj-5ta fibroblasts in extract assay, 24 h incubation. Data are the mean ± SD; the viability of non-treated (intact) cells was taken as 100%.

**Figure 11:**
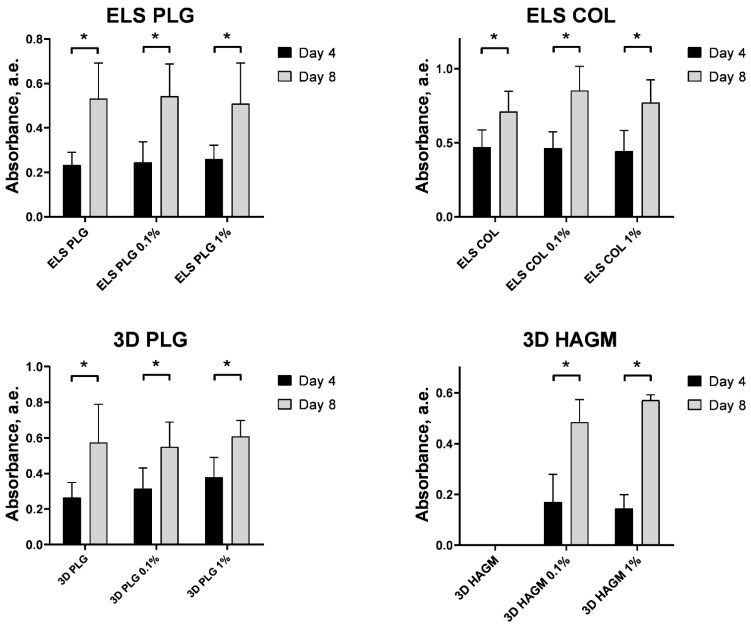
The growth of Bj-5ta fibroblasts on the UCNPs-loaded scaffolds, 4 and 8 days of incubation. Data are the mean ± SD, * *p* < 0.05 in Mann–Whitney U test.

We evaluated the cytotoxicity of the obtained scaffolds in vitro using Bj-5ta fibroblasts in extract and contact assays. For the extract assay, we measured the cytotoxicity of the medium after 24 h of conditioning in the presence of the UCNP-loaded scaffolds. We did not find any significant cytotoxicity of UCNP-loaded scaffolds in the extract assay in comparison to the blank polymer either for 0.1% or 1% UCNPs loading (Figure 10). In another experiment, we evaluated the cell amount on days 4 and 8 using an MTT assay to demonstrate the cell growth on the surface of the scaffolds. It was found that the number of cells significantly (*p* < 0.05 in Mann–Whitney U test) increased within the cultivation regardless of the material used and UCNP content (Figure 11). The impregnated UCNPs did not influence the cell growth that confirmed their biocompatibility in vitro.

**Figure 12:**
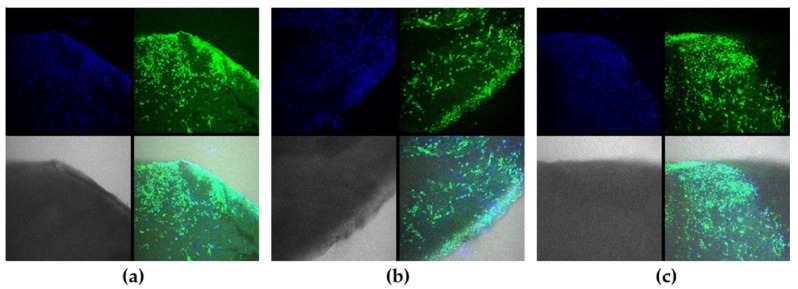
Confocal images of ELS COL (**a**), ELS COL 0.1% (**b**), and ELS COL 1% (**c**) scaffolds cultured with Bj-5ta fibroblasts, 8 days of incubation. Green is for calcein AM staining (live cells), blue is for Hoechst 33342 staining (cell nuclei).

The colonization of the scaffold surface with fibroblasts within 8 days of incubation was demonstrated by confocal microscopy (Figure 12, Figures S7 and S8). The bright calcein AM staining confirmed high cell viability; the pattern of colonization differed in dependence of the material used for scaffolds generation. Thus, we found homogenous cell distribution for collagen- and PLGA-based scaffolds, while cell aggregates were typical for 3D HAGM scaffolds. No significant differences in cell distribution were found for pure polymers and UCNP-loaded scaffolds.

## 3. Materials and Methods

### 3.1. Materials

Polylactic-co-glycolic acids (PLGA) Purasorb PDLG7507 (PURAC Biochem, Netherlands) with an inherent viscosity midpoint of 0.7 dL/g and a lactic-to-glycolic-acid monomer ratio of 75:25 and type I collagen (COL, Nearmedic Plus LLC, Moscow, Russia) were used as polymeric materials for electrospinning. PLGA, hyaluronic acid glycidyl methacrylate (synthesized at the Federal Research Scientific Center “Crystallography and Photonics” RAS), polyethylene glycol diacrylate (PEGDA, Sigma-Aldrich, St. Louis, MO, USA), flavin mononucleotide (Pharmstandard, Moscow, Russia), and triethanolamine (Sigma-Aldrich, St. Louis, MO, USA) were used as initial materials for 3D printing. Highly volatile 1,1,1,3,3,3-hexafluoroisopropanol (HFIP, 99%, P&M-Invest, Moscow, Russia) and tetraglycol (Sigma Aldrich, St. Louis, MO, USA) were used to mix the polymeric solutions for scaffold fabrication. As a cross-linking agent, 1,4-butanediol diglycidyl ether (BDDGE, ≥95%, Sigma Aldrich, St. Louis, MO, USA) was used, and isopropanol (99%, Ekos-1, Moscow, Russia) was used for collagen chemical stabilization. Reagents for synthesis of UCNPs (Y_2_O_3_, Yb_2_O_3_, Er_2_O_3_, CF_3_COOH:H_2_O = 3:1, (CF3COO)Na, 1-octadecene, and oleic acid) were purchased from Sigma Aldrich, St. Louis, MO, USA.

### 3.2. Modification of Hyaluronic Acid with Glycidyl Methacrylate

Sodium hyaluronate (M_n_ = 100 kDa), glycidyl methacrylate (GMA) were purchased from Sigma Aldrich, St. Louis, MO, USA. N,N-dimethylformamide (≥99.8%) and acetone (≥99.7%) were purchased form Chimmed, Moscow, Russia. Penicillin–streptomycin (5.000 U/mL and 5.000 µg/mL respectively) was purchased from PanEco, Moscow, Russia. Amphotericin B (5.000 µg/mL) was purchased from JSC “Sintez”, Kurgan, Russia. Modification of hyaluronic acid with glycidyl methacrylate (GMA) was carried out similarly to the method described in [58]. First, 1 g of sodium hyaluronate (salt form of HA) was completely dissolved in 100 mL of deionized water. In order to suppress possible growth of microorganisms, the HA solution was supplemented with 500 μL of penicillin–streptomycin (5000 U/mL of penicillin G and 5000 μg/mL of streptomycin) and 128 μL of amphotericin B (5 mg/mL). Then, 70 mL of N,N-dimethylformamide and 12 mL of GMA were added to start the reaction. The reaction proceeded for 3 days under continuous stirring at 30 °C. The resulting product, hyaluronic acid modified with glycidyl methacrylate (HAGM), was isolated by precipitation in 7-fold excess of acetone. The product was purified by dissolving the precipitate in distilled water and subjecting it to dialysis against a 10-fold excess of distilled water for 4 days, with daily changes of water. The purified HAGM was then frozen and lyophilized in FreeZone Freeze Dry System (Labconco, Kansas City, MO, USA). The degree of substitution of HA disaccharide units with conjugated vinyl groups was measured according to the protocol of colorimetric reaction from [59] and was defined as 31%.

### 3.3. Synthesis of Upconversion Nanoparticles

The synthesis of β-NaYF_4_:Yb^3+^:Er^3+^ (20% Yb, 2% Er) nanoparticles with undoped NaYF_4_ shells was carried out by the thermolysis of precursors in high-boiling (290–310 °C) solvents (oleic acid and 1-octadecene) that we described earlier [47]. Briefly, a mixture of oxides Y_2_O_3_, Yb_2_O_3_, Er_2_O_3_ was boiled in the CF_3_COOH:H_2_O = 3:1 system until dissolved. Then, 2 eq (CF3COO)Na, 15 mL of 1-octadecene (≥99%), and 15 mL of oleic acid (≥99%) were added to the obtained trifluoroacetates (CF_3_COO)_3_Y, (CF_3_COO)_3_Yb, and (CF_3_COO)_3_Er. To decompose trifluoroacetates and form in situ β-NaYF_4_:Yb^3+^:Er^3+^ nanocrystals, the flask was placed in Rose’s alloy heated 360 °C. After 30 min, the flask was removed from the Rose’s alloy, and 15 mL of 1-octodecene was added for rapid cooling. The particles were washed with isopropanol and centrifuged at 6000 rpm for 30 min. The reaction was monitored by changing the light transmission of the reaction mixture and the photoluminescence of the reaction product. The synthesized nanoparticles were covered with a crystalline inert NaYF_4_ shell according to the method described above.

### 3.4. Electrospinning

PLGA and COL compositions for electrospinning were prepared by dissolving polymers in HFIP in a ratio of 9 wt.% and 4 wt.%, respectively. Then, BDDGE (0.5 to 3 wt.%) was added to the original collagen composition as a cross-linking agent. The formation of thin polymer fibers and porous films was carried out on a custom-build experimental setup for electrospinning (ELS) of polymer solutions (Figure 2a and Figure S9). The polymer solution in HFIP was fed with a pump through a polyethylene tube into a stainless steel needle (diameter = 0.36 mm) connected to a metal electrode, to which a voltage varying from 10 to 25 kV was applied. After the solvent had evaporated, the electrically deflected polymer filaments were deposited onto a collecting electrode (collector) covered with a grounded aluminum foil. After preliminary optimization of the electrospinning modes, the formation of ELS PLGA and ELS COL scaffolds occurred at the following parameters: voltage ΔV = 20 kV, solution supply rate v = 2 mL/h, distance between the tip of the needle and the collector l = 12 cm.

### 3.5. Chemical Cross-Linking of Collagen

The electrospun collagen scaffolds were placed into isopropanol solution containing 15 wt.% BDDGE at a temperature of 37 °C for 6 days to increase their mechanical properties. The pH level was 5.9 for the whole period of time.

### 3.6. Antisolvent 3D Printing

PLGA composition for anti-solvent 3D printing was prepared by dissolving the polymer in tetraglycol in a ratio of 10 wt.%. The formation of 3D PLGA scaffolds was carried out by the method of anti-solvent 3D printing [32], based on layer-by-layer application of a polymer solution to a substrate followed by its curing by phase separation upon contact with aqueous medium. The PLGA solution was loaded into the custom-build extruder of the 3D printer (Figure 2b and Figure S10) and applied through a needle with an inner diameter of 0.2 mm to the bottom of a Petri dish filled with distilled water. The fabrication of 3D structures was carried out layer-by-layer in accordance with a 3D computer model (7 mm diameter, 0.5 mm height, 85% filling density, and ~180 μm layer thickness). The extrusion was carried out at an average printing speed of 1 mm/s, and solution flow rate was 0.05 µL/s. After printing, the scaffolds were immersed in distilled water at 25 °C for 24 h to ensure final curing.

### 3.7. Extrusion 3D Printing with Simultaneous Photocuring

The initial photopolymerizable composition (PPC) consisted of an aqueous solution of 19.7 wt.% HAGM, 4.9 wt.% PEGDA, 0.1 wt.% flavin mononucleotide, and 0.5 wt.% triethanolamine. For the manufacture of scaffolds based on hyaluronic acid (3D HAGM), we used an original three-dimensional extrusion printer of our own design (Figure 2c and Figure S11). The principle of its operation is based on the layer-by-layer application of PPC along a trajectory determined by a custom-written 3D computer model with simultaneous photo-curing by laser radiation (λ = 445 nm).

### 3.8. Microscopy

The microstructure and surface morphology of the experimental samples were studied using a Phenom ProX scanning electron microscope (Phenom, Eindhoven, the Netherlands). The accelerating voltage used for imaging was typically 10 kV.

### 3.9. Analysis of Photoluminescent Properties of Polymer Scaffolds

Free UCNPs were resuspended in *n*-hexane (99%, Ekos-1, Russia) with the final concentration of 35 mg/mL and placed onto a glass slide; the hexane was evaporated at room temperature. The photoluminescence spectra of the samples excited by a continuous semiconductor laser with a wavelength of 976 nm were recorded using a Fluorolog-3 spectrofluorometer (Horiba Jobin Yvon, Longjumeau, France). For visualization of UCNPs, a custom-built imaging system was used [60], equipped with a Raylase scanner head (Raylase, Wessling, Germany). The signal is recorded using a highly sensitive EMCCD camera (Raptor Photonics Incorporated, Larne, UK). An LDD-10 semiconductor laser (JSC Semiconductor Devices, Saint Petersburg, Russia) with a fiber output was used as an excitation source at a wavelength of 976 nm. The laser radiation intensity was 200 mW/cm^2^.

### 3.10. Light Transmittance

To measure the polymer materials transparency, 10 µL of the initial COL and PLGA compositions for electrospinning was dropped on the bottom of the Petri dish and left under normal conditions until the solvent completely evaporated to obtain a film 200 µm thick. The same amount of HAGM PPC was dropped on the bottom of the Petri dish and photo-cured by laser radiation (λ = 445 nm). The transmission spectra of the obtained films were measured using a Cary 50 spectrophotometer (Varian, Walnut Creek, USA).

### 3.11. Release of UCNPs In Vitro

A sample of the UCNP-loaded matrix weighing 1.7 mg was placed in 0.25 mL of PBS and kept in a shaker (Orbital Shaker-Incubator ES-20, BioSan, Riga, Latvia) at a temperature of 37 °C. The intensity of UCNPs released into the aqueous medium was measured on days 1, 3, and 5. UCNPs spectra were recorded in PBS (Eco-service, Saint Petersburg, Russia) and distilled water. UCNPs were added at a concentration of 0.7 mg/mL in both media.

### 3.12. Cytotoxicity

The scaffolds were washed in sterile PBS (pH 7.4) and placed in complete DMEM culture medium for 24 h in a CO_2_ incubator (37 °C, 5% CO_2_). The obtained scaffold-conditioned medium was added to Bj-5ta fibroblasts previously planted into 96-well plates (5 × 10^3^ cells per well), and the plates were transferred to a CO_2_ incubator for another 24 h. Cell viability was assessed via MTT assay (3 h incubation with 0.5 mg/mL 3-(4,5-dimethylthiazol-2-yl)-2,5-diphenyltetrazolium bromide followed by dissolution in 100 µL DMSO and optical absorbance measurement at 565 nm); the viability of non-treated cells was taken as 100%.

### 3.13. Cultivation of Fibroblasts on The Surface of Scaffolds

Scaffolds were sterilized in 70% ethanol for 30 min, washed in PBS (pH 7.4), and kept in complete DMEM culture medium for 24 h in a CO_2_ incubator (37 °C, 5% CO_2_). Then, the scaffolds were placed on the non-adhesive (agarose) surface into 24-well plates, and 5 × 10^4^ fibroblasts were added to each well in 1 mL of complete DMEM. The plates were placed in a CO_2_ incubator, and a complete replacement of the medium was carried out every 2–3 days. Cell growth on the surface of the scaffolds was assessed using an inverted light microscope. The number of cells was quantitatively measured by MTT assay on days 4 and 8 as described above.

### 3.14. Confocal Imaging

Scaffolds were stained with calcein AM (50 µM, 15 min), fixed in 4% formaldehyde (room temperature, 30 min) and stained with Hoechst 33342 dye (50 µM, 15 min). Then, the stained samples were washed in PBS (pH 7.4) three times and studied using a Leica TCS SPE confocal fluorescent system (Leica, Wetzlar, Germany), 405 nm and 488 nm excitation.

## 4. Conclusions

In our study, two types of synthetic- (PLGA) and natural-origin (COL and HAGM) polymer scaffolds were used to produce 8 types of UCNP-loaded samples using two types of manufacturing methodologies. The optical photoluminescent properties of UCNP-loaded scaffolds were analyzed using an advanced imaging technique. Scaffolds impregnated with core/shell β-NaYF_4_:Yb^3+^, Er^3+^/NaYF_4_ nanoparticles were visualized with a high optical contrast when excited by laser irradiation at 976 nm. It was found that the spectrum of UCNPs incorporated into the scaffolds differed from intact nanoparticles, but this did not interfere with their visualization. Additionally, the spectra of nanoparticles were sensitive to their microenvironment, mainly depending on the type of polymer used. These finding provide an opportunity not only of simple visualization, but the real time analysis of the scaffolds state and microenvironment. The experiments have shown that the release of UCNPs from the scaffold into the environment induces R/G ratio changes, thereby allowing real-time monitoring of scaffold degradation. We speculate that in vivo monitoring can become possible after adapting the visualization system for separate registration of photoluminescence at wavelengths of 544 and 658 nm. The developed UCNP-impregnated scaffolds were not cytotoxic and provided a surface for the cell attachment, proliferation, and colonization necessary for tissue repair. In summary, demonstrated viability of the developed approach to technological platform for “smart scaffold” fabrication based on UCNP-loaded bioresorbable polymer structures impregnated with UCNPs, providing the desired photoluminescent, biochemical, and mechanical properties for intravital visualization and monitoring of their behavior and tissue/scaffold interaction in real time.

## Data Availability

Not applicable.

## Supplementary Materials

The following supporting information can be downloaded at: https://www.mdpi.com/article/10.3390/molecules27196547/s1, Figure S1: Electrospun collagen scaffolds characterization: normalized photoluminescence spectra of the 0.1 and 1 mg of UCNPs per 100 mg of polymer included in ELS COL scaffolds (**a**); photograph of ELS COL scaffolds with 0 (control), 0.1 and 1 mg of UCNPs per 100 mg of polymer at 976 nm (**b**); Figure S2: Electrospun polylactic-co-glycolic acids scaffolds characterization: normalized photoluminescence spectra of the 0.1 and 1 mg of UCNPs per 100 mg of polymer included in ELS PLGA scaffolds (**a**); photograph of ELS PLGA scaffolds with 0 (control), 0.1 and 1 mg of UCNPs per 100 mg of polymer at 976 nm (**b**); Figure S3: 3D printed polylactic-co-glycolic acids scaffolds characterization: normalized photoluminescence spectra of the 0.1 and 1 mg of UCNPs per 100 mg of polymer included in 3D PLGA scaffolds (**a**); photograph of 3D PLGA scaffolds with 0 (control), 0.1 and 1 mg of UCNPs per 100 mg of polymer at 976 nm (**b**); Figure S4: 3D printed HAGM scaffolds characterization: normalized photoluminescence spectra of the 0.1 and 1 mg of UCNPs per 100 mg of polymer included in 3D HAGM scaffolds (**a**); photograph of 3D HAGM scaffolds with 0 (control), 0.1 and 1 mg of UCNPs per 100 mg of polymer at 976 nm (**b**); Figure 5: Dependence of transmittance of the polymer films; Figure S6: Characterization of upconversion core/shell β-NaYF4:Yb3+:Er3+ (NaYF4:Yb3+) nanoparticles in aqueous media: Dependence of the ratio of the intensity of the red luminescence band at a wavelength of 658 nm to that of the green one at a wavelength of 544 nm on the power density of exciting laser radiation for UCNPs in different media (**a**); normalized photoluminescence spectra of the UCNPs and the UCNPs included in ELS COL scaffolds (**b**); Ratio of the intensity of the red peak at 658 nm to the intensity of the green one at a wavelength of 544 nm of 1% UCNPs and UCNPs released from ELS COL scaffolds into PBS (**c**); Figure S7: Confocal images of 3D HAGM 0,1% (**a**) and 3D HAGM 1% (**b**) scaffolds cultured with Bj-5ta fibroblasts, 8 days incubation. Green is for Calcein AM staining (alive cells), blue is for Hoechst 33342 staining (cell nucleuses); Figure S8: Confocal images of ELS PLG (**a**), ELS PLG 0,1% (**b**), and ELS PLG 1% (**c**) scaffolds cultured with Bj-5ta fibroblasts, 8 days incubation. Blue is for Hoechst 33342 staining (cell nucleuses), no Calcein AM staining. Scale bar is 400 µm; Figure S9: Custom-designed electrospinning set-up. 1—Polymer solution supply system, 2—Syringe, 3—Stainless steel capillary, 4—Aluminum foil collector, 5—High voltage supply; Figure S10: 3D printer for antisolvent printing. 1—Stepper motor, 2—Needle, 3—Syringe, 4—Pump, 5—PC, 6—Samples, 7—Petri dish, 8—Motor guides; Figure S11: Extrusion 3D printer with laser sources at a wavelength of 445 nm. 1—Power supply for laser modules, 2—Printer power supply, 3—Semiconductor lasers, 4—Stepper motor (for moving the extruder piston), 5—Extruder, 6—Extruder nozzle, 7—Arduino controller, 8—Stepper motor (for moving desktop along the Z axis), 9—Stepper motor (to move the extruder along the X axis), 10—Substrate, 11—Worktable, 12—Chamber with a filter, 13—Stepper motor (to move the desktop along the Y axis); Table S1: Lifetime of photoluminescence of UCNPs.

## Sample Availability

Samples of the compounds are available from the authors.
