# Supplementary material for: Natural and Synthetic Polymer Scaffolds Comprising Upconversion Nanoparticles as a Bioimaging Platform for Tissue Engineering
Source: Molecules. 2022 Oct 3;27(19):6547. doi: 10.3390/molecules27196547 (PMC9573624; doi:10.3390/molecules27196547)
Supplement: Supplementary file 1 [file molecules-27-06547-s001.zip › molecules-1910095-supplementary.pdf]

Natural and synthetic polymer scaffolds comprising upconversion nanoparticles as a bioimaging platform for tissue engineering

Ekaterina M. Trifanova[1], Maria A. Khvorostina[1], Aleksandra O. Mariyanats[1], Anastasia V. Sochilina[1], Maria E. Nikolaeva[2], Evgeniy V. Khaydukov[1], Roman A. Akasov[1*] and Vladimir K. Popov[1].

[1] Institute of Photon Technologies of Federal Scientific Research Centre "Crystallography and Photonics" of Russian Academy of Sciences, Moscow, Russia;
[2] NPF Syntol, Moscow, Russia.
* Correspondence: roman.akasov@gmail.com

Appendix A

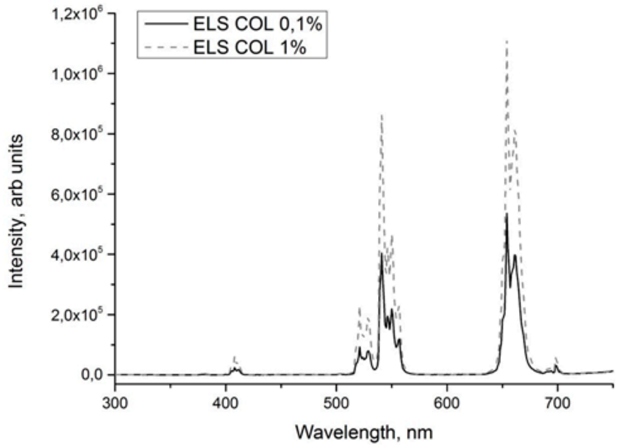

(a)

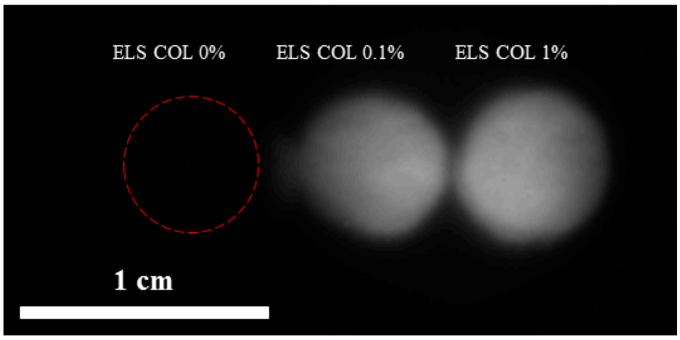

(b)

Figure S1. Electrospun collagen scaffolds characterization: normalized photoluminescence spectra of the 0.1 and 1 mg of UCNPs per 100 mg of polymer included in ELS COL scaffolds (**a**); photograph of ELS COL scaffolds with 0 (control), 0.1 and, 1 mg of UCNPs per 100 mg of polymer at 976 nm (**b**).

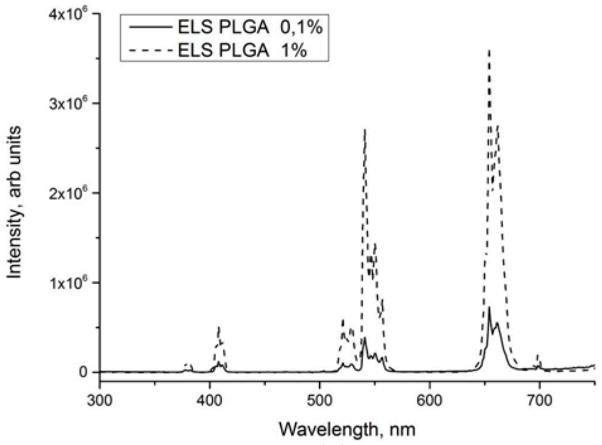

(a)

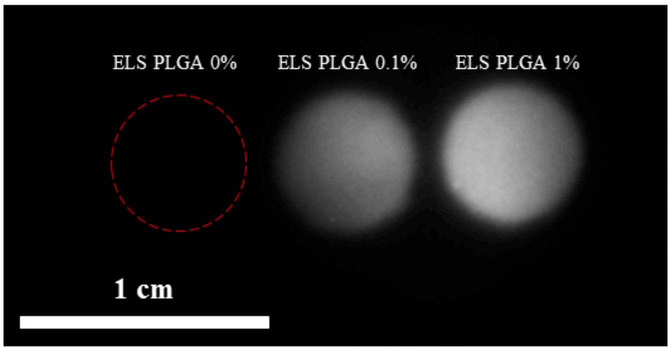

(b)

Figure S2. Electrospun polylactic-co-glycolic acids scaffolds characterization: normalized photoluminescence spectra of the 0.1 and 1 mg of UCNPs per 100 mg of polymer included in ELS PLGA scaffolds (**a**); photograph of ELS PLGA scaffolds with 0 (control), 0.1 and, 1 mg of UCNPs per 100 mg of polymer at 976 nm (**b**).

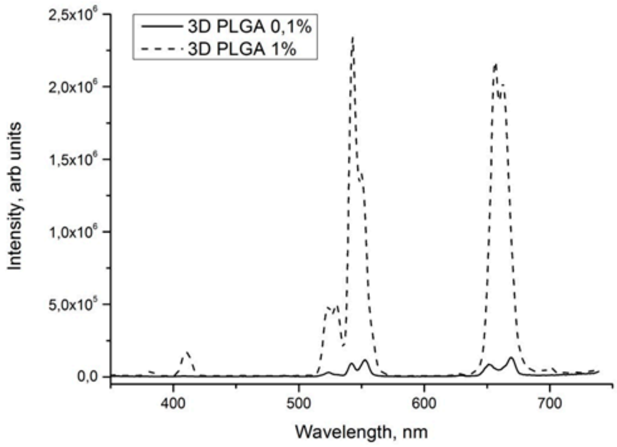

(a)

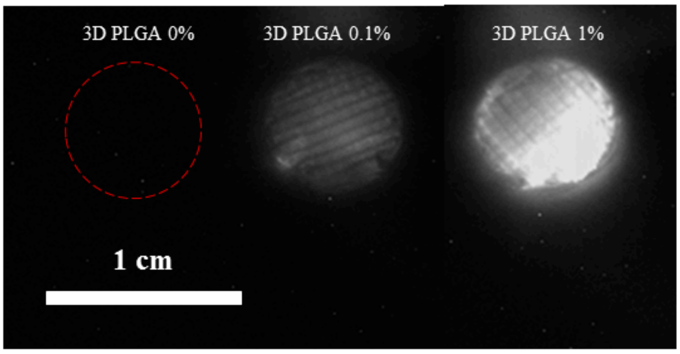

(b)

Figure S3. 3D printed polylactic-co-glycolic acids scaffolds characterization: normalized photoluminescence spectra of the 0.1 and 1 mg of UCNPs per 100 mg of polymer included in 3D PLGA scaffolds (**a**); photograph of 3D PLGA scaffolds with 0 (control), 0.1, and 1 mg of UCNPs per 100 mg of polymer at 976 nm (**b**).

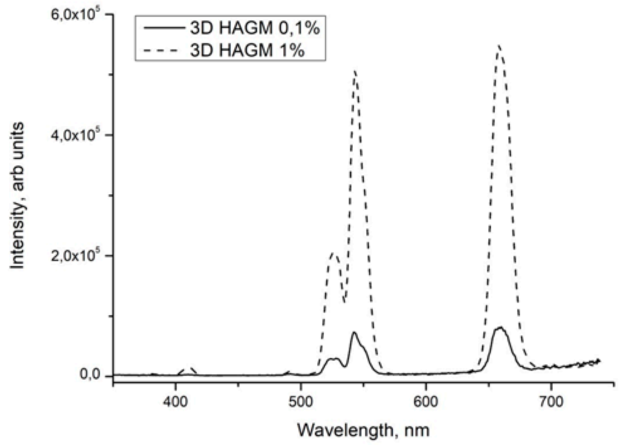

(a)

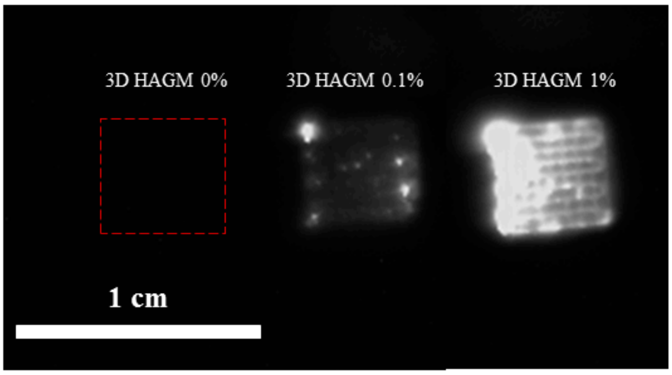

(b)

Figure S4. 3D printed HAGM scaffolds characterization: normalized photoluminescence spectra of the 0.1 and 1 mg of UCNPs per 100 mg of polymer included in 3D HAGM scaffolds (**a**); photograph of 3D HAGM scaffolds with 0 (control), 0.1, and 1 mg of UCNPs per 100 mg of polymer at 976 nm (**b**).

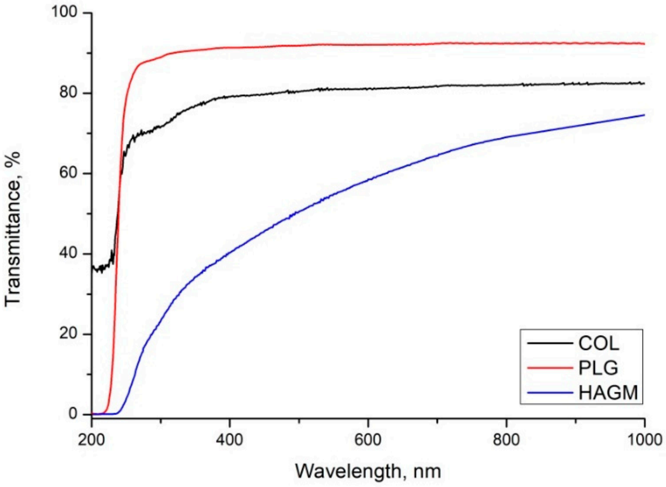

Figure S5. Dependence of transmittance of the polymer films.

Table S1. Lifetime of photoluminescence of UCNPs.

Photoluminescence lifetime, ms						
**Wavelength, nm**	**UCNPs**	**UCNPs in water**	**UCNPs in ELS COL**	**UCNPs in ELS PLGA**	**UCNPs in 3D PLGA**	**UCNPs in 3D HAGM**
**409**	0.21 ± 0.01	0.19 ± 0.01	0.15 ± 0.01	0.14 ± 0.01	0.20 ± 0.01	0.13 ± 0.01
**525**	0.32 ± 0.02	0.22 ± 0.02	0.22 ± 0.02	0.18 ± 0.02	0.24 ± 0.02	0.17 ± 0.01
**544**	0.38 ± 0.01	0.23 ± 0.03	0.24 ± 0.02	0.22 ± 0.02	0.31 ± 0.02	0.25 ± 0.02
**658**	0.33 ± 0.01	0.31 ± 0.02	0.32 ± 0.01	0.31 ± 0.03	0.35 ± 0.03	0.25 ± 0.02

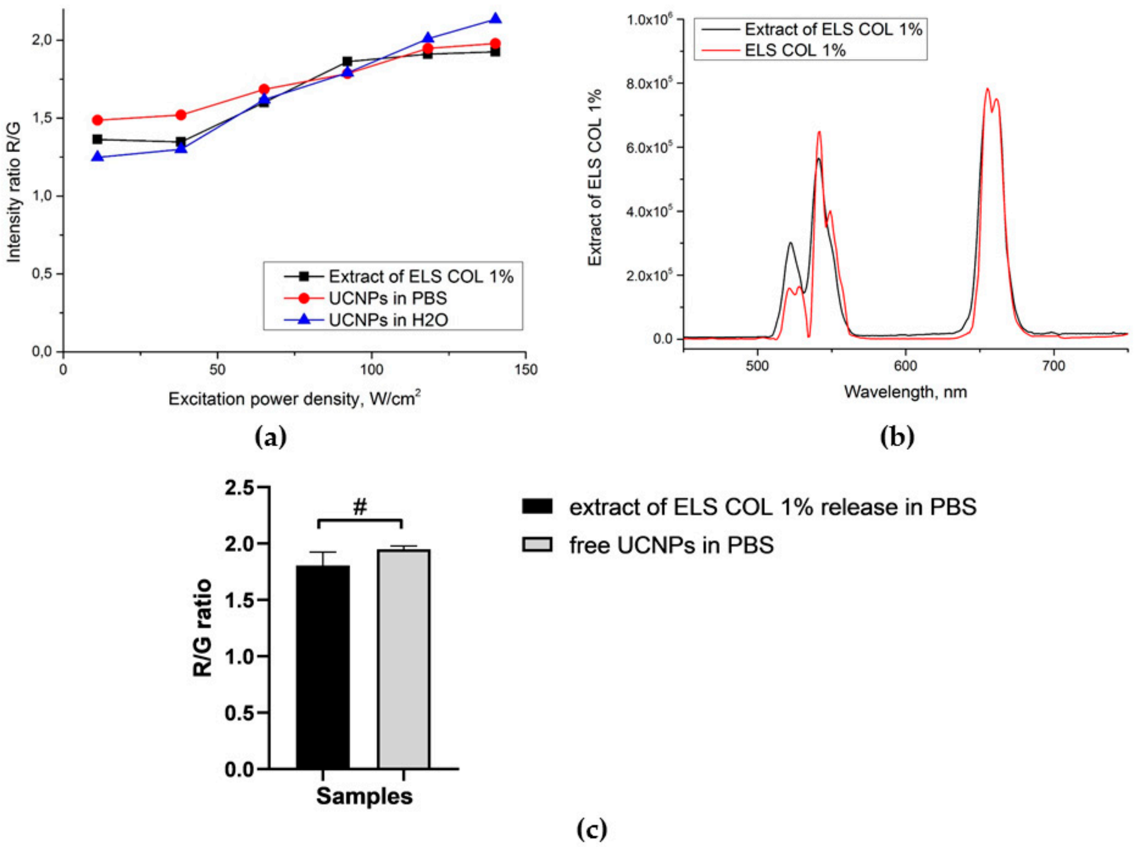

Figure S6. Characterization of upconversion core/shell β-NaYF4:Yb3+:Er3+ (NaYF4:Yb3+) nanoparticles in aqueous media: Dependence of the ratio of the intensity of the red luminescence band at a wavelength of 658 nm to that of the green one at a wavelength of 544 nm on the power density of exciting laser radiation for UCNPs in different media (**a**); normalized photoluminescence spectra of the UCNPs and the UCNPs included in ELS COL scaffolds (**b**); Ratio of the intensity of the red peak at 658 nm to the intensity of the green one at a wavelength of 544 nm of 1% UCNPs and UCNPs released from ELS COL scaffolds into PBS (**c**).

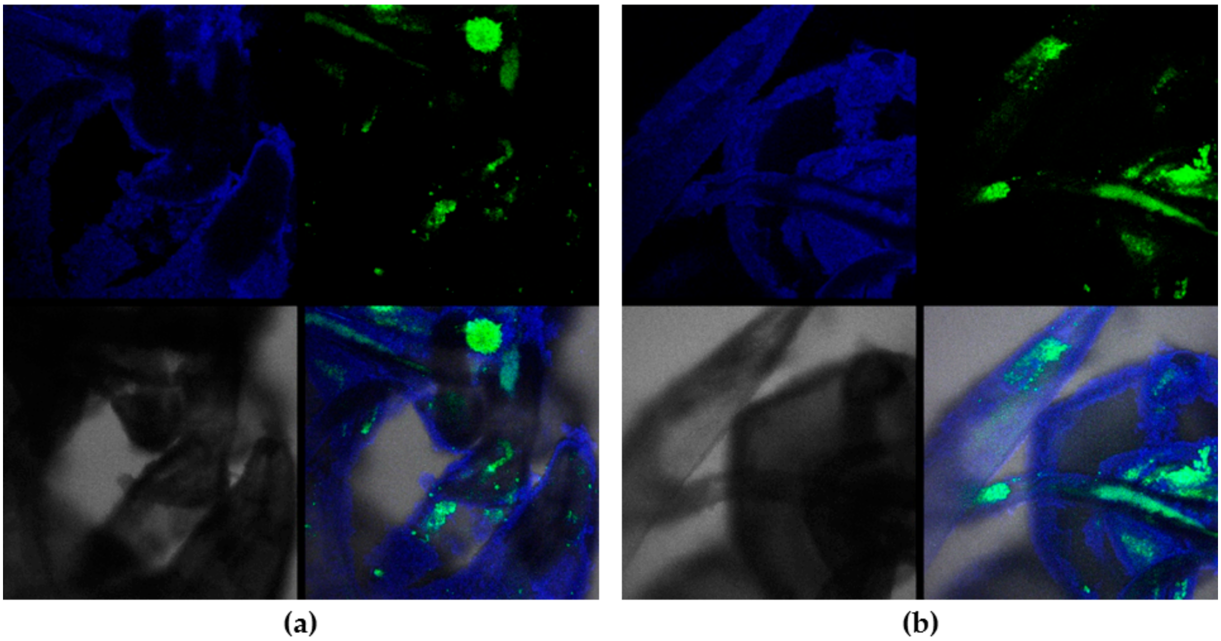

Figure S7. Confocal images of 3D HAGM 0,1% (**a**) and 3D HAGM 1% (**b**) scaffolds cultured with Bj-5ta fibroblasts, 8 days incubation. Green is for Calcein AM staining (alive cells), blue is for Hoechst 33342 staining (cell nucleuses).

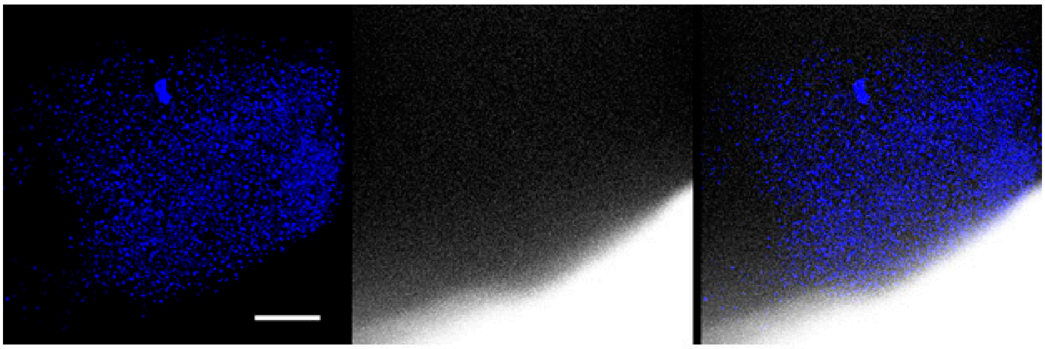

(a)

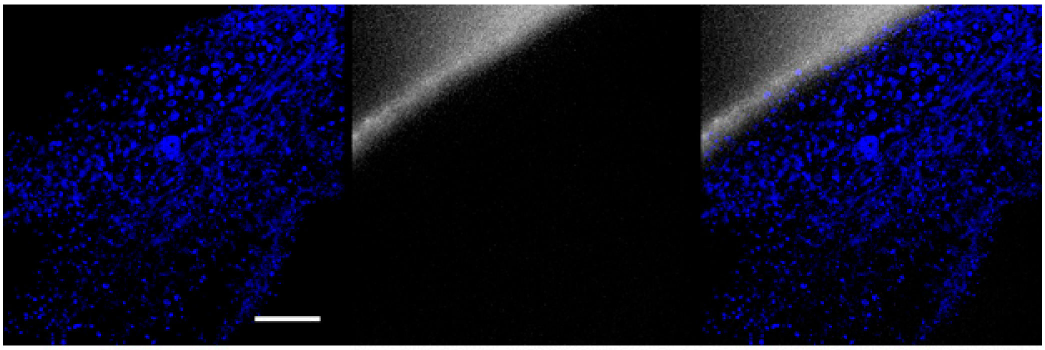

(b)

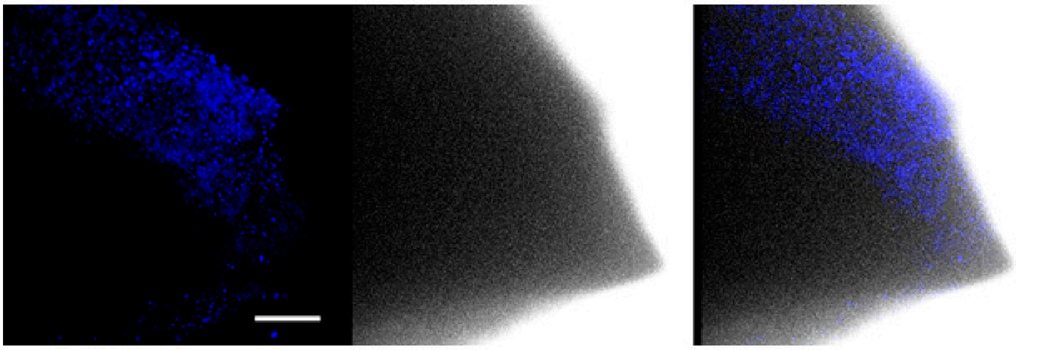

(c)

Figure S8. Confocal images of ELS PLG (**a**), ELS PLG 0,1% (**b**), and ELS PLG 1% (**c**) scaffolds cultured with Bj-5ta fibroblasts, 8 days incubation. Blue is for Hoechst 33342 staining (cell nucleuses), no Calcein AM staining. Scale bar is 400 μm.

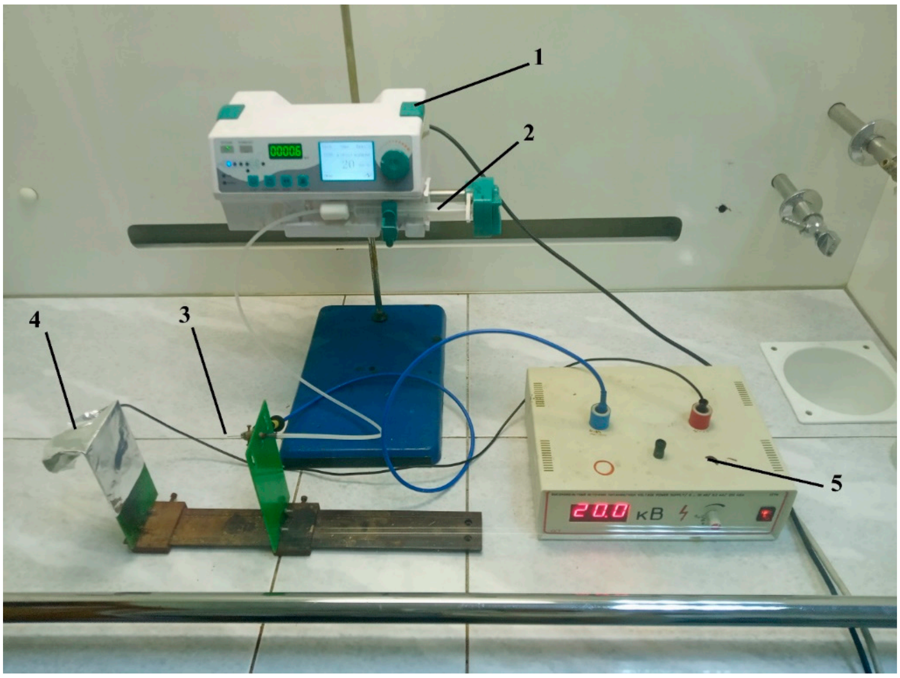

Figure S9. Custom-designed electrospinning set-up. 1 - polymer solution supply system, 2 – syringe , 3 - stainless steel capillary, 4 – aluminum foil collector, 5 – high voltage supply.

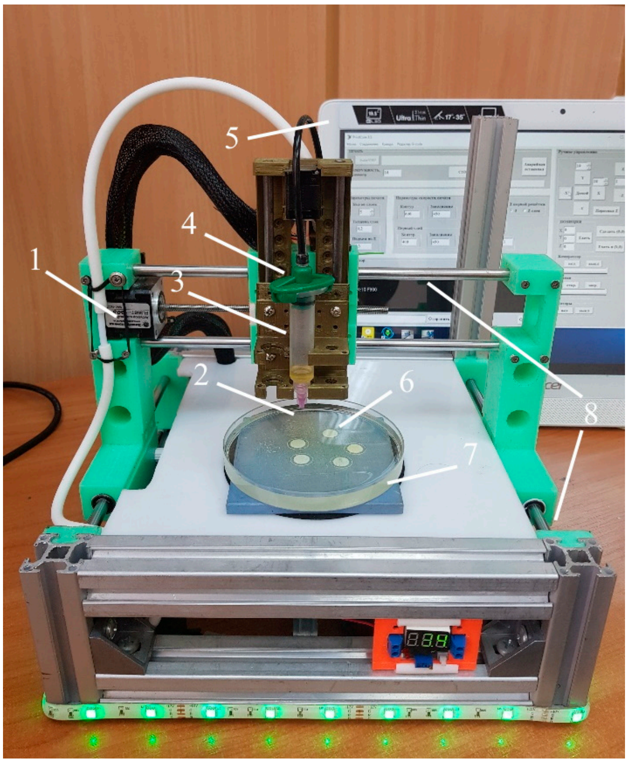

Figure S10. 3D printer for antisolvent printing. 1 – stepper motor, 2 – needle, 3 – syringe, 4 – pump, 5 – PC, 6 – samples, 7 – Petri dish, 8 – motor guides.

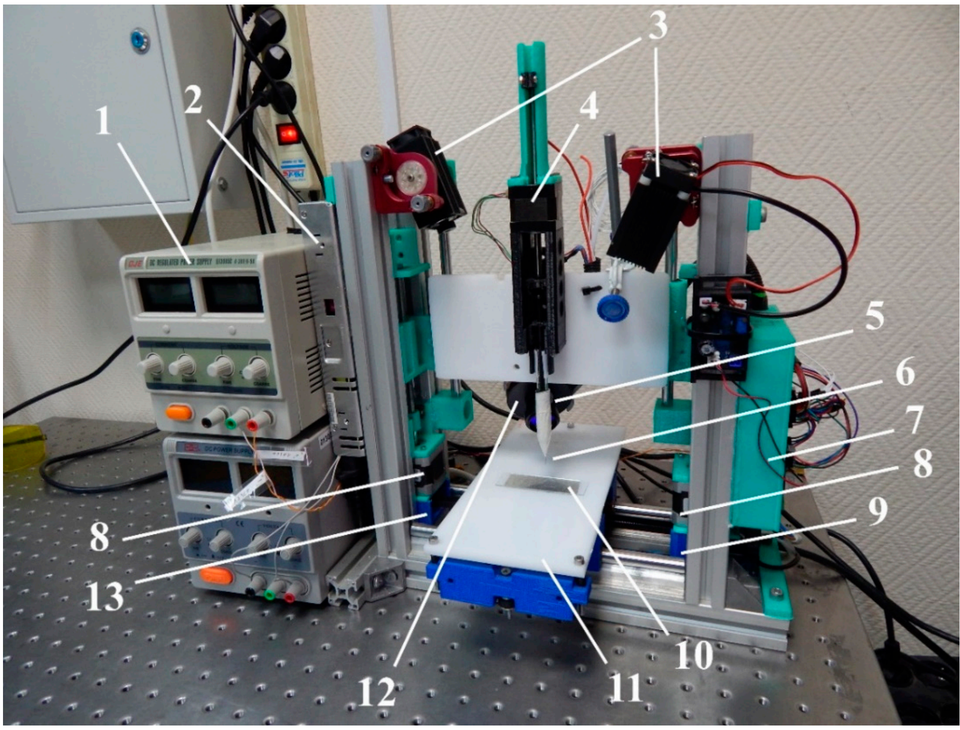

Figure S11. Extrusion 3D printer with laser sources at a wavelength of 445 nm. 1 - power supply for laser modules, 2 - printer power supply, 3 - semiconductor lasers, 4 - stepper motor (for moving the extruder piston), 5 - extruder, 6 - extruder nozzle, 7 - Arduino controller, 8 - stepper motor (for moving desktop along the Z axis), 9 - stepper motor (to move the extruder along the X axis), 10 - substrate, 11 - worktable, 12 - chamber with a filter, 13 - stepper motor (to move the desktop along the Y axis).
